# Family Ties and Late-Life Well-Being: Evaluating Patterns Across Sociocultural and Welfare State Policy Contexts

**DOI:** 10.1093/geroni/igaf122.1313

**Published:** 2025-12-31

**Authors:** Deborah Carr, Nekehia Quashie

## Abstract

Older adults’ well-being is powerfully shaped by the instrumental, socioemotional, and material supports received by spouses and adult children. However, the extent to which family ties enhance specific dimensions of well-being may vary across national contexts. Cultural factors, including normative expectations regarding family support, and policy contexts, including the level of public supports for older adults’ economic and physical well-being, may moderate the effects of family support on late-life well-being. These four papers use cross-national data and quantitative methods to evaluate the role of sociopolitical contexts in the lives of older adults. Wang and Yang use data from the Health and Retirement Study (HRS) and China Health and Retirement Longitudinal Study (HRS) and find that marital histories affect loneliness and social participation differently for older adults in the U.S. vs. China. Jessee and Carr use Survey of Health, Ageing and Retirement in Europe (SHARE) data to examine linkages between parent-child disconnectedness and older adult mental health, and find pronounced effects in Southern Europe, where familism is a pervasive value. Azar, Wahrendorf and Cheng use SHARE data to document linkages between intergenerational financial support and older adults’ depressive symptoms, and find patterns moderated by welfare regime characteristics. Mahmoud and Carr use SHARE data to examine associations between marital status and place of death, and find that the protective effects of marriage, especially for women, differ across contexts, although marital history is unrelated to place of death in Nordic states with generous welfare regimes. Implications for policy and practice are highlighted.

